# A novel cellular automaton approach for modeling genotypic and phenotypic heterogeneity in cell systems

**DOI:** 10.1140/epjs/s11734-026-02186-1

**Published:** 2026-02-24

**Authors:** Simon Syga, Josué Manik Nava-Sedeño, Andreas Deutsch

**Affiliations:** 1 Center for Interdisciplinary Digital Sciences, Department for Information Services and High Performance Computing, TUD Dresden University of Technology, 01062 Dresden, Germany; 2 Department of Mathematics, Faculty of Sciences, Universidad Nacional Autónoma de México, Circuito Exterior, Ciudad Universitaria, 04510 Mexico City, Mexico

## Abstract

Cellular automaton models have long been used to study cellular processes, but may be challenging for incorporating synchronization, migratory, and high-density effects. In this work, we introduce an extension of the classic lattice-gas cellular automata, a framework which allows to consider changes in cell numbers, cell–cell interactions, migration, and evolution of genotypic and phenotypic heterogeneity. To demonstrate the utility of our approach, we consider a growing population of cells whose genotype—passed on at birth from the mother cell and subject to stochastic mutations—determines their individual proliferation rates. Using spatial simulations, we show that the model exhibits traveling-wave invasion patterns, where the fastest-growing cells accumulate at the leading edge, accelerating population expansion. We predict this behavior using a mathematical analysis based on a mean-field assumption.

**Supplementary Information:**

The online version contains supplementary material available at https://doi.org/10.1140/epjs/s11734-026-02186-1.

## Introduction

Mathematical modeling plays a crucial role in understanding complex physical and biological systems. A central question is how local interactions among individuals give rise to global behaviors and macroscopic patterns—a topic of sustained interest across disciplines. Among various modeling approaches, cellular automata (CA) offer a particularly versatile framework. These discrete dynamical systems represent space, time, and states on a grid where each site evolves through deterministic or stochastic rules. Because processes like birth, death, and phenotypic change are naturally implemented in this format, CA models are especially suited for studying cellular dynamics.

Originally introduced by von Neumann to explore self-replication, CA have been widely applied in fields ranging from fluid dynamics and pattern formation to tumor growth [1–5]. Lattice-gas cellular automata (LGCA), a variant of CA, incorporate cell velocities explicitly, enabling simultaneous modeling of transport and reaction processes. Initially developed for fluid simulations [1, 6], LGCA have since been applied to biological systems, including cell migration, excitable media, and tumor development [4, 7, 8]. Their fully discrete structure efficiently captures stochasticity, local interactions, and heterogeneity in complex environments [4, 9, 10]. Regarding fluid dynamics applications, LGCA have evolved further into the so-called *lattice-Boltzmann methods*, though their basic principles of discretization and separation of the dynamics into collision and streaming steps are conserved [11].

Despite their strengths, classical LGCA models face limitations. They assume indistinguishable cells, struggle with high-density behaviors, and generally omit evolutionary changes, such as genotype or phenotype variation. These issues can introduce artifacts and limit their applicability to real biological systems. In response, several LGCA extensions have been proposed to better capture individual-level variability.

Here, we present an extension called evolutionary LGCA (evo-LGCA), recently used to study tumor heterogeneity driven by evolving phenotypic plasticity [12]. As an example, we examine the spatial expansion of a heterogeneous cell population undergoing Darwinian evolution. Each cell’s proliferation rate is inherited with stochastic mutations. Simulations reveal traveling-wave expansion, with faster-growing cells dominating the invasion front. This behavior is predicted analytically using a mean-field approximation. Our results offer a discrete modeling framework that links individual cellular traits to large-scale tumor growth, providing insights into evolution, heterogeneity, and invasion dynamics.

## Lattice-gas cellular automata

Unlike classical cellular automata (CA), which describe cell presence or absence per site, LGCA incorporate *velocity channels*, allowing explicit modeling of directed cell movement. This framework has been used to study collective migration, excitable media, tumor invasion, and other biological processes.

LGCA are defined on a regular *d*-dimensional lattice $\mathcal {L}$. Each node $\textbf{r}\in \mathcal {L}$ has *b* nearest neighbors determined by lattice geometry: in 1D, b=2, in 2D, b=4 (square) or b=6 (hexagonal). Nodes are connected to neighbors via *velocity channels*: unit vectors pointing to adjacent nodes. Additionally, each node may contain $a\in \mathbb {N}_0$ rest channels, representing zero-velocity states. Velocity channels represent the possible velocities a cell can have when moving, while rest channels help representing cells that decide to remain immobile. Furthermore, the number *a* of rest channels helps controlling the cellular diffusion coefficient, as explained in detail in [5]. Effectively, the lattice $\mathcal {L}$ discretizes space, while the set of velocity and rest channels discretizes momentum. Thus, the LGCA framework effectively discretizes the system’s phase space [11].

Each node has a state $\textbf{s}\in \mathcal {E}$, where $\mathcal {E}=\mathcal {O}^C$, C=a+b, where $\mathcal {O}$ is the set of possible occupancies for each channel. The set $\mathcal {O}$ represents the possible number and/or properties of cells moving in each of the allowed discrete directions defined by velocity and rest channels. All state spaces $\mathcal {O}$ must contain an element representing an empty channel, to indicate that there is no cell of any kind moving in a certain direction (or equivalently, no stationary cell if considering rest channels). For example, spatial regions devoid of cells are modeled by nodes with only empty velocity and rest channels. Spatial regions where cells are moving as a cohesive unit would have all but one channel empty. In classic LGCA, $\mathcal {O}=\{0,1\}$, i.e., each channel is either empty or occupied by a single cell: an *exclusion principle* analogous to the Pauli principle in quantum mechanics. Hence, the node state is $\textbf{s}=\left( s_0,s_1,\ldots ,s_{C-1}\right) ^T\in \{0,1\}^C,$ and the total number of cells at a node is $n(\textbf{s})=\sum _{j=0}^{C-1}\textbf{s}_j$. Every lattice site can contain up to *C* cells. This make LGCA *mesoscopic models*, which do not strictly model the dynamics of single cells, but do not model population densities, either. The *lattice spacing* is typically set so that the measure of each lattice site matches that of *C* biological cells.

A node’s update depends on the states in its *neighborhood* $\mathcal {N}$. The most common choice is the von Neumann neighborhood $\mathcal {N}_N$, comprising a node and its direct neighbors; for a 2D square lattice, $\left| \mathcal {N}_N\right| =5$ [10]. The state of the neighborhood is denoted by $\textbf{s}_{\mathcal {N}}\in \mathcal {E}^{|\mathcal {N}|}$. The neighborhood represents the set of nearby cells that are able to interact with cells in a certain node. Thus, the neighborhood defines the local environment during cell interactions.

**Fig. 1:**
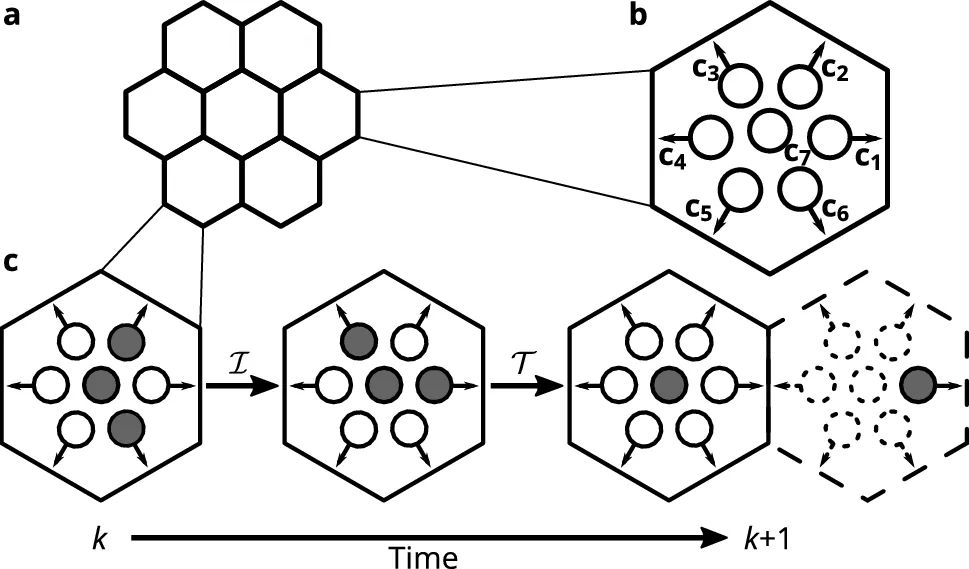
**The classic LGCA model dynamics:** **a** the LGCA model is defined on a regular lattice, here a hexagonal lattice; **b** each lattice node has *b* velocity channels allowing movement to the nearest-neighbor nodes and *a* rest channels modeling no movement. There are six velocity channels due to the hexagonal lattice and one rest channel. Each channel can be occupied (gray) or empty (white); **c** during one discrete time step k→k+1, each node is updated simultaneously according to a stochastic interaction step $\mathcal {I}$ and a subsequent deterministic transport step $\mathcal {T}$. During the interaction step, cells might be removed, added, and rearranged within the node’s velocity channels. In this example, the particles are redistributed among channels. Subsequently, during the transport step, cells in velocity channels are propagated to the respective nearest-neighbor node (dotted lattice node; only the neighbor to the right is shown)

Time evolves in discrete steps $k\in \mathbb {N}$ via parallel application of the update rule $\mathcal {R}=\mathcal {T}\circ \mathcal {I}$, composed of a stochastic interaction step $\mathcal {I}$ followed by a deterministic transport step $\mathcal {T}$ (Fig. 1). The splitting of the dynamics into two distinct steps is analogous to that of particle fluid dynamics algorithms, from which LGCA originate. In every time step, particles undergo collisions, which change their velocities, followed by streaming, where particles move in the direction of their new velocities [11]. These correspond to the interaction and transport steps in LGCA, respectively. The full configuration is a stochastic process $\boldsymbol{\eta }:\mathcal {L}\times \mathbb {N}\rightarrow \mathcal {E}$, with the local and neighborhood configurations denoted $\boldsymbol{\eta }(\textbf{r}, k)$ and $\boldsymbol{\eta }_{\mathcal {N}}(\textbf{r}, k)$, respectively. During the interaction step, a new configuration $\boldsymbol{\eta }'(\textbf{r},k)$ is sampled using a Markovian operator $\mathcal {I}\left( \textbf{s}_\mathcal {N}\right) :\mathcal {E}^{|\mathcal {N}|}\rightarrow \mathcal {E}$, according to the transition probability $P(\textbf{s} \rightarrow \textbf{s}' \mid \textbf{s}_{\mathcal {N}})$ which encodes all cellular processes, e.g., birth, death, motility changes due to cellular cues, or response to the environment. It has been shown that these transition probabilities may be explicitly obtained as the probability distributions of Brownian, interacting cells [13]. Often, $\mathcal {I}$ is composed of sequential rules for each biological mechanism. For example, a proliferation-death-diffusion model applies birth/death updates followed by intra-node redistribution. In the transport step, cells move to neighboring nodes along their velocity channels

1 $$\begin{aligned} \eta _j(\textbf{r},k+1)=\eta _j'(\textbf{r}-\textbf{c}_j,k), \end{aligned}$$

where $\eta _j(\textbf{r},k)$ is the occupation of the channel *j* at node $\textbf{r}$ and time *k*, and $\textbf{c}_j$ is the corresponding channel. This *microdynamical equation* defines the LGCA’s spatiotemporal evolution. The microdynamical equation conveys that a cell’s velocity at the next time step is the same as it was in the previous time step before moving from the neighboring node but after the interaction step. For finite lattices, appropriate *boundary conditions* must be specified, e.g., periodic, reflective, or absorbing, to reflect biological constraints and experimental design [10].

### Individual-based lattice-gas cellular automata (IB-LGCA)

One major limitation of classic LGCA is that cells are all identical, and thus indistinguishable, since only the presence or absence of cells is represented by the state space. However, in many applications, it is necessary to track individual particles, e.g., to study the statistics of movement [14]. Particles could also be heterogeneous with individual characteristics influencing their behavior [15]. To overcome this issue, in IB-LGCA, each particle carries a label, called property *p*, from a property set $\mathcal {P}$. The state space is extended to

2 $$\begin{aligned} \mathcal {E} = \mathcal {P}^C, \end{aligned}$$

where a special default property $p_0$ represents an empty channel, that is, the absence of a cell.

This generalization enables IB-LGCA to model cells with different phenotypes or fitness levels by identifying each phenotype or fitness level with a different label. IB-LGCA are useful and flexible, but implementing the interaction rule can be computationally expensive due to the large number of possible node configurations. For example, in a node with five channels and four cells (occupied channels), a cell-preserving rule would need to consider only five different configurations in the classic LGCA, but 5!=120 configurations in IB-LGCA, if all cells had different properties. Moreover, the framework still implements the exclusion principle (one cell per channel), which means that, in the limit $n({\textbf {s}})\rightarrow C$, cells are always moving, and their directions are uniformly distributed (each channel is occupied). In many practical applications, this behavior is not desired. For example, instead of moving in every direction uniformly, biological cells in a monolayer show jamming behavior, where cell speed decreases with increasing cell density [16].

### Boson lattice-gas cellular automata (BLGCA)

BLGCA removes the exclusion principle by allowing multiple cells to occupy the same channel, thus removing high-density artifacts while remaining computationally efficient. BLGCA have been used to model populations of compressible swarming cells [17], and to study the dynamics of tumor formation in highly motile, adhesive cancer cell populations [18]. The name was chosen in analogy to bosons, particles that can be in the same quantum state simultaneously. The state space is modified to

3 $$\begin{aligned} \mathcal {E} = \mathbb {N}_0^C, \end{aligned}$$

where each channel contains an integer amount of cells instead of a Boolean number. Although the change of the state space in BLGCA seems small, it allows us to define the interaction step in a more efficient, simplified way for many applications. If we assume that the interaction rule is such that each cell is independently assigned a channel, then we may define channel probabilities $T_i({\textbf {s}}_{\mathcal {N}}): \mathcal {E}^{\vert {\mathcal {N}}\vert } \mapsto [0, 1]$, representing the probability that a single cell is assigned the *i*th channel during the interaction step, that is, the probability that a cell would change its velocity to the velocity $\textbf{c}_i$. Channel probabilities depend on the states of neighboring nodes, since velocity changes are assumed to occur due to interactions with neighboring cells or the local environment. Clearly, for all cell-conserving interactions, the probabilities of the channels must sum up to one, i.e., $\sum _{i=0}^b T_i({\textbf {s}}_{\mathcal {N}}) = 1$. Therefore, to define mass-conserving interactions in BLGCA, it is sufficient to specify the channel probabilities $T_i({\textbf {s}}_{\mathcal {N}})$. As a simple example, unbiased cell movement could be modeled with $T_i = 1/C$. BLGCA are particularly useful for modeling biological systems where crowding effects and high-density interactions play a role, and when individuals are significantly elastic. However, in BLGA, as in classic LGCA, cells are undistinguishable, which does not allow cells to be tracked or to behave uniquely.

## Evolutionary lattice-gas cellular automata (evo-LGCA)

**Fig. 2:**
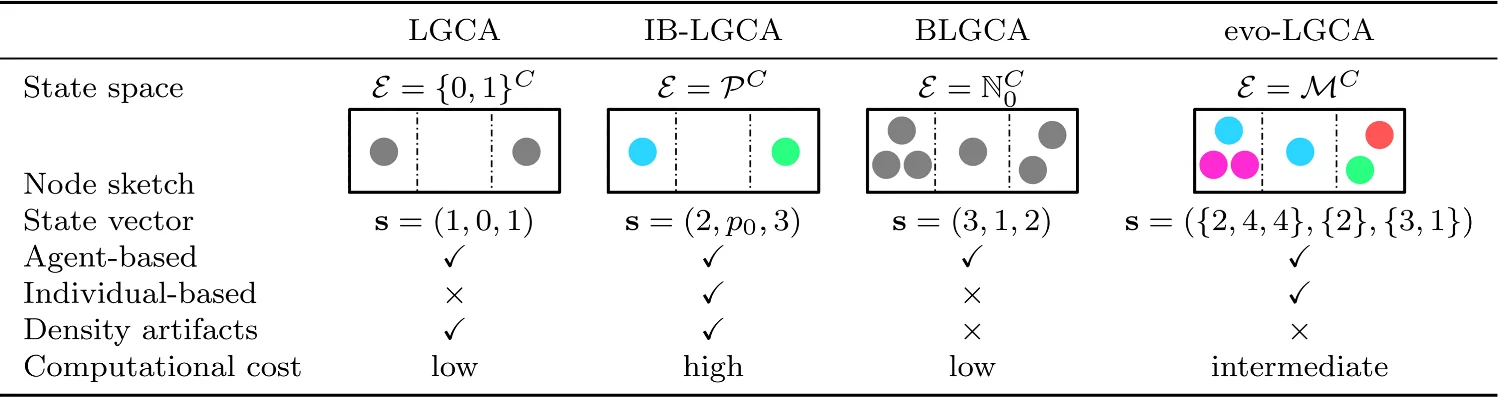
Characteristics of different LGCA frameworks. All LGCAs are defined on a discrete spatial lattice, and every lattice node has velocity channels; for example, a one-dimensional lattice can have three velocity channels: left, right, and rest. The channel occupation set defines the LGCA’s state space and subtype (see example sketches in the third row, separation between channels is indicated with a dash-dotted line)

Classical LGCA, IB-LGCA, and BLGCA, provide powerful frameworks for modeling discrete, migrating cell populations. However, these frameworks suffer from either artifacts at high density or the lack of cell identity. To address these limitations simultaneously, we introduce the *evolutionary lattice-gas cellular automaton* (evo-LGCA), a modeling framework that extends LGCA by allowing individual-based interactions while lifting the exclusion principle, thereby enabling the study of heterogeneous and evolving populations (Fig. 2). The name underscores its biological application rather than its origins. See Supplementary Information for in-depth mathematical framework details.

### Concept and state space

The state space is defined as

4 $$\begin{aligned} \mathcal {E} = \mathcal {M}^C, \end{aligned}$$

where each channel holds a *multiset* $s_i \in \mathcal {M}$ of particle properties, allowing multiple cells with equal or different attributes to occupy the same channel. A multiset differs from a conventional set in that it allows multiple occurrences of the same element, making it a natural representation for a population of individuals with distinct but possibly overlapping characteristics.

For instance, consider a scenario where particles represent biological cells, and their property $p \in \mathcal {P}$ encodes their proliferation rate. If a channel has a state $s_i = \{p_1, p_1, p_2, p_3\}$, it means that it contains two cells with property $p_1$, one with property $p_2$, and one with property $p_3$. The absence of cells is indicated by the empty set $s_i = \emptyset$.

Each individual in evo-LGCA is assigned a property $p \in \mathcal {P}$ from a flexible property set $\mathcal {P}$. This set can represent various attributes, such as proliferation rate, migratory behavior, or phenotypic state. For example, in a biological application, $\mathcal {P}$ could include discrete states, such as $\{\text {migratory}, \text {proliferative}\}$ or continuous states representing a metabolic rate. The flexibility of the property set makes evo-LGCA a versatile framework for modeling heterogeneous populations undergoing selection and mutation.

A key advantage of this approach is that it combines the ability to track individual identities and assign unique behaviors to every property (as in IB-LGCA) with the capacity to model high-density populations (as in BLGCA). This makes evo-LGCA particularly suited for studying scenarios where spatial structure and evolutionary changes interact, such as tumor growth or bacterial population dynamics.

### Dynamics

Fundamentally, the dynamics in the evo-LGCA framework works like in classical LGCA and its extensions defined above: Dynamics proceeds in discrete time steps that are split into two substeps, a stochastic interaction step and a deterministic propagation step.

#### Interactions conserving mass and properties

As in BLGCA, mass and property-conserving interactions can be defined via a transition probability of the following form, assuming that each cell is assigned a channel independently with a channel occupation probability $T_i(p, {\textbf {s}}_\mathcal {N})$ that can depend on the property *p* of the cell.

The channel probabilities $T_i(p, {\textbf {s}}_{\mathcal {N}}): \mathcal {P} \times \mathcal {E}^{\vert {\mathcal {N}}\vert } \mapsto [0, 1]$ represent the probability that a cell with property *p* is assigned a channel *i* during the interaction step. If the interaction is mass and property-conserving, we have $\sum _{i=0}^{C-1} T_i(p, {\textbf {s}}_{\mathcal {N}}) = 1$ for all $p \in \mathcal {P}$. Note that evo-LGCA and BLGCA coincide if all cells are identical, i.e., have the same transition probability.

#### Birth and death interactions

If cells undergo proliferation, apoptosis, or phenotypic change, then cell numbers and/or properties are not conserved, and the interaction step must add these processes on top of mass and property-conserving interactions. To simulate apoptosis, one can independently remove a cell with property *p* on the *i*th channel with probability $\delta _i(p)$, iterating over all cells to decide whether they die or not.

Simulating proliferation is slightly more complicated. Every cell can proliferate every time step with probability $$\alpha _i(p)$$. If daughter cells mutate, they obtain a different property than that of the mother cell. We must therefore define the probability mass or probability density $f(p^{\prime },p)$ that a daughter cell has a probability $p^{\prime }$. Finally, daughter cells are placed in velocity channels with probability $$T_i \in [0, 1], i = 0, 1, \dots , C-1$$.

## Expansion of a heterogeneous cell population under Darwinian evolution

As a prime application example, the evo-LGCA framework facilitates the study of emergent evolutionary dynamics of heterogeneous cell populations. We define a simple model of individual cells that proliferate, move, die, and change their proliferation rate through mutation. Using computer simulations, we show how, starting from a localized cell population, the population expands like a propagating wave with increasing wave speed, caused by an accumulation of fast-growing cells at the wave front. Under certain approximations, we derive a macroscopic equation for the cell density and the wave speed depending on cell motility and proliferation rate, which is validated through simulations.

### Model definition

The model describes a population of individual cells characterized by a proliferation rate $p = \alpha \in \mathcal {P} = [0,1]$. Each cell undergoes migration, proliferation, death, and mutation according to the following rules:

- **Migration:** Each cell performs an unbiased random walk with a diffusion constant D>0.
- **Proliferation:** A cell at node $\textbf{r}$ proliferates with probability $\alpha (1 - n(\textbf{r})/K)$, where *K* is the local carrying capacity, i.e., the maximum allowed number of cells per node, analogously to logistic growth models.
- **Death:** Each cell dies with a constant probability δ.
- **Mutation:** Upon proliferation, the daughter cell inherits the mother’s proliferation rate with probability $1 - p_\mu$. With probability $p_\mu$, the daughter’s proliferation rate is randomly altered by a mutation, with probability density $f(\alpha _i, \alpha _j)$ centered around the cell progenitor’s proliferation rate.

In the model, the stochastic interaction step is split into two operators: First, a birth-and-death reaction operator removes dying cells and adds new ones. Next, all cells are randomly redistributed between local channels following a mass-conserving operator. To simulate unbiased movement and to tune the motility (or equivalently, the diffusion coefficient) of cells, we define equal occupation probabilities for all velocity channels, and give a special weight to the occupation of rest channels. Thus, cells may prefer to move or to remain stationary depending on this weight. The probability of a cell being placed in a velocity channel is $T_i = 1/Z$ for i=0,⋯,b-1, and the probability for the rest channel is $T_{b} = \frac{\exp \gamma }{Z}$, where *Z* is a normalization constant $Z:= b + \exp \gamma$, and the parameter $\gamma \in \mathbb {R}$ is chosen in such a way that the diffusion constant in the macroscopic limit is equal to *D*.

### Simulation setup and observables

Simulations were conducted in a setup that mimics invasion in one dimension. To this end, we employ a two-dimensional square lattice with reflecting (Neumann) boundary conditions and lattice dimensions $$L_x \times L_y = 500 \times 10$$. To allow for enough simulation time until the boundary is reached by the population and to reveal the evolutionary dynamics, we used a large number of nodes along the *x*-axis. Moreover, we added ten nodes along the *y*-axis to improve the simulation statistics without increasing the computational costs of the simulation too much. For analysis, we project the system along the *y*-direction to obtain the density profile $$\Psi (x,t):= \left\langle {\Psi (x, y, t)}\right\rangle _y$$ and treat it as one-dimensional. Initially, cells are considered to be genotypically homogeneous and located at the main tumor body. Thus, the simulation was initialized with cells only at the left boundary with an initial proliferation rate $$\alpha _0 = 0.2$$ and a local equilibrium density $$\Psi _0 = 1 - \delta / \alpha _0$$. For the other parameters, we choose: $$\delta = 0.01, p_\mu = 1, D=0.14$$ and, to simulate a generic mutation process, we defined the probability density of the proliferation rate to mutate from α to $\alpha^{\prime }$, $$f(\alpha , \alpha ')$$, as a Gaussian distribution^1^ centered at α with variance $$\sigma ^2=10^{-4}$$. Key observables measured in the simulations include the following:

- **Wavefront position** $x_{\text {front}}(t)$: Defined as the first spatial position where the density drops below a threshold $\Psi _{\text {front}}:= 0.1 \Psi _0$. This observable gives a measure of the spatial spread of the cell population at a given time *t*.
- **Mean proliferation rate** $\langle \alpha \rangle (x,t)$: Averaged over all cells at each spatial position *x* and along the *y*-axis. This observable allows tracking the spatial evolution of the population’s proliferation rates.
- **Wave speed** *c*(*t*): Computed from the temporal evolution of $x_{\text {front}}(t)$. This quantifies the speed with which cells colonize free space away from the tumor.

**Fig. 3:**
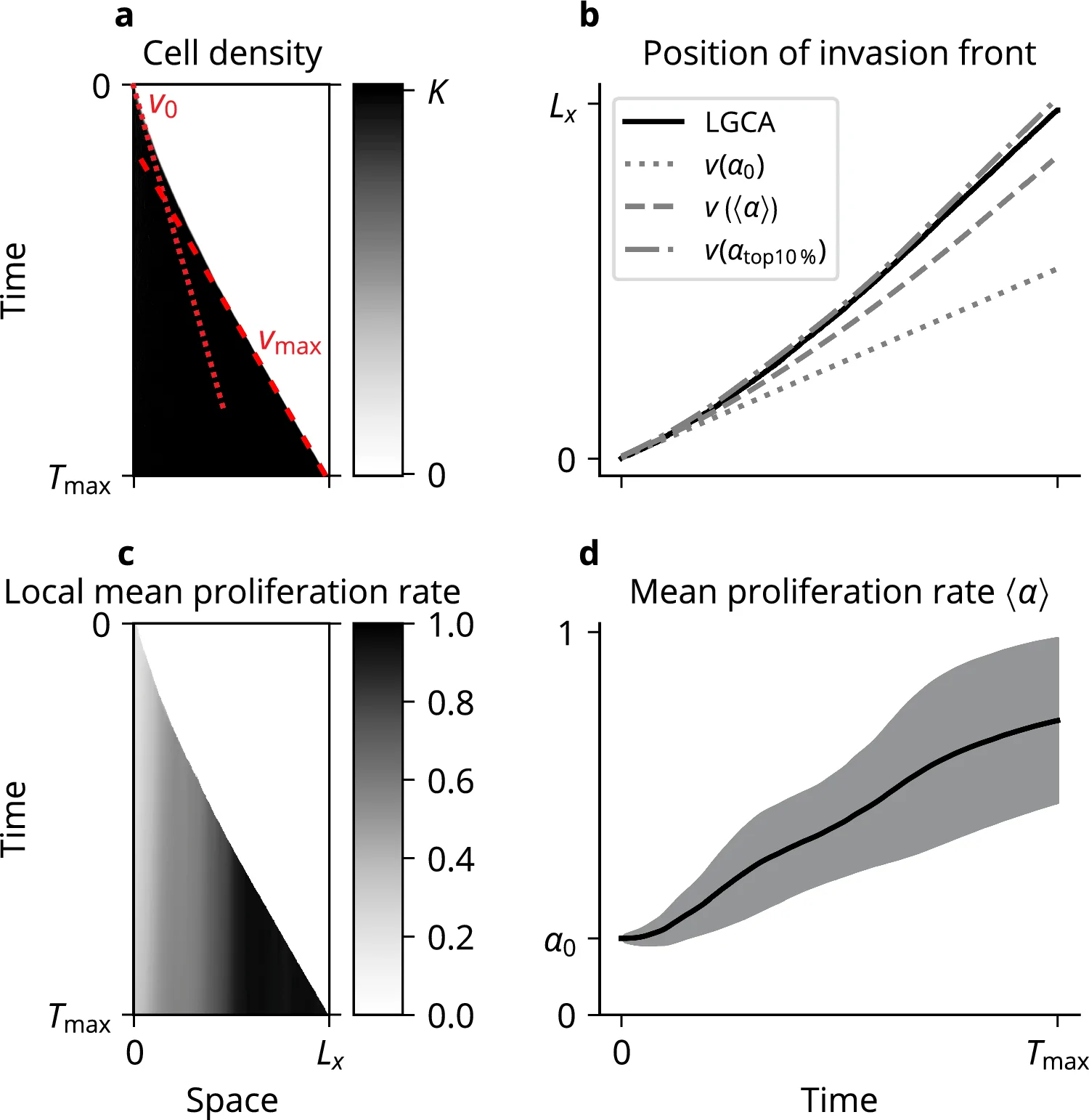
**Analytic and simulation results of evolutionary dynamics in growing populations.** **a** Spatiotemporal cell density. The population spreads like a traveling wave with a wave speed that increases over time. Red lines show wave fronts if the wave speed were constant. **b** The position of the wavefront increases non-linearly, indicating a time-dependent wave speed. A mean-field approximation assuming that the wave speed depends on the proliferation rate of the 10% fastest-growing cells (gray dash-dotted line) can predict the agent-based simulation (black line). Predictions based on the initial proliferation rate or the mean value differ significantly from the value of the LGCA simulations (gray dotted/dashed lines). **c** Spatiotemporal distribution of the mean proliferation rate. The fastest-growing cells, indicated by darker gray shades, are located near the wavefront. **d** Mean proliferation rate over time. Gray bands indicate one standard deviation. The mean and standard deviation increase over time. See text for parameter values

### Mathematical and computational analysis

To analyze the spatial expansion of the population, we derive a macroscopic description of the density Ψ(x,t), representing the total number of cells at spatial position *x* and time *t* (see Supplementary Information). The evo-LGCA model leads to a generalized Fisher-KPP equation

5 $$\begin{aligned} \partial _t \Psi = D \partial _x^2 \Psi + \langle \alpha \rangle \Psi (1 - \Psi ) - \delta \Psi , \end{aligned}$$

where $\langle \alpha \rangle$ is the mean proliferation rate at position *x* and time *t*. This equation predicts traveling-wave solutions, where the population expands with an evolving front speed $c(\left\langle {\alpha }\right\rangle )$.

Analogously to the classical Fisher-KPP equation [19, 20] the wave speed is given by

6 $$\begin{aligned} v(\alpha ) = 2 \sqrt{D (\langle \alpha \rangle - \delta )}. \end{aligned}$$

Since $\langle \alpha \rangle$ is not constant, but rather changes due to selection and mutation, the wave speed changes over time. Note that the wave speed is only defined when $\langle \alpha \rangle >\delta$. Otherwise, the population will become extinct instead of propagating as a wave.

Computer simulations confirm these analytic predictions: As seen in Fig. 3, the population invades empty space as a wave, with the wavefront advancing non-linearly, accelerating over time. It can be observed that, on average, the fastest-growing cells accumulate at the invasion front, while less proliferating cells linger at the back, near the boundary, leading to the formation of a spatial gradient of proliferation rates. Since the front velocity (Eq. 6) depends on the time-dependent average proliferation rate $\left\langle \alpha \right\rangle$, in principle, it would be necessary to obtain an expression for $\left\langle \alpha \right\rangle (t)$. Instead, to fit the wavefront speed, $\left\langle \alpha \right\rangle$ was obtained directly from simulation observables (e. g. Fig. 3d). Three options were considered: assigning $\left\langle \alpha \right\rangle$ the value of the initial proliferation rate $\alpha _0$, the mean proliferation rate of the population, or the average value of the 10% fastest-growing cells, i.e., the average of the 10% higher values of α in the population. Comparing these different approaches, the best predictor is the mean proliferation rate of the top 10% of cells at the front.

## Discussion

We introduced the evolutionary lattice-gas cellular automaton (evo-LGCA), a novel extension of LGCA capable of modeling heterogeneous cell populations with dynamic genotypes and phenotypes, without artifacts at high densities. Other LGCA variants include the following:

- Classic LGCA: Assumes indistinguishable cells with exclusion constraints; suited for low-density, homogeneous populations; highly efficient.
- Individual-based (IB) LGCA: Tracks unique cell identities with exclusion; enables heterogeneity but at higher computational cost.
- Boson LGCA (BLGCA): Allows high densities without exclusion, but assumes identical, evolutionarily static cells; computationally efficient.
- Evolutionary LGCA (evo-LGCA): Tracks unique cells without exclusion; suited for dense, evolving populations with moderate computational demands.

To demonstrate the evo-LGCA framework, we modeled a population of proliferating cells undergoing mutations in their proliferation rate. Simulations revealed traveling-wave dynamics, spontaneous spatial sorting of faster-growing genotypes at the front, and an accelerating wavefront. This can be observed in breast cancer, non-small cell lung cancer, and high-grade gliomas leading to the development of a novel biomarker based on the relative position of the metabolic hotspot in the tumor [21, 22]. These behaviors were captured by a generalized Fisher-KPP mean-field approximation, validating the model’s ability to link individual-level evolution to macroscopic invasion dynamics. This also extends previous continuum approaches by incorporating mutation-selection processes into the framework [21, 23].

Our findings agree with recent models of tumor invasion, supporting evo-LGCA as a robust tool for capturing evolutionary dynamics in expanding populations. However, some limitations remain. We assumed a Gaussian distribution of mutational effects, whereas real fitness landscapes may be more complex [24, 25]. The model also focuses solely on proliferation-based fitness, omitting trade-offs such as migration-proliferation dynamics [12, 26, 27]. Moreover, our simulations addressed one-dimensional expansion; higher-dimensional and heterogeneous tissue geometries warrant future exploration [28].

Evo-LGCA offers a quantitative, discrete framework for studying spatial evolution across biological contexts. Potential applications include tumor progression [29], bacterial adaptation in structured environments [30], and ecological invasions under evolutionary pressure [31].

## Supplementary Information

Below is the link to the electronic supplementary material.

Supplementary file 1 (pdf 296 KB)

## Data Availability

The authors declare that the data supporting the findings of this study are available within the paper, its supplementary information files, and in the repository mentioned within the code availability statement.

## Footnotes

1 We choose a Gaussian distribution for simplicity rather than biological realism.

## Declarations

### Code availability

A general evo-LGCA code as well as the specific model simulated in this work can be accessed at https://github.com/sisyga/biolgca.
