# Supplementary material for: A novel cellular automaton approach for modeling genotypic and phenotypic heterogeneity in cell systems
Source: Eur Phys J Spec Top. 2026 Feb 24;235(31):8869–77. doi: 10.1140/epjs/s11734-026-02186-1 (PMC13743661; doi:10.1140/epjs/s11734-026-02186-1)
Supplement: Supplementary file 1 [file 11734_2026_2186_MOESM1_ESM.pdf]

Supplementary information: A novel cellular automaton approach for modeling genotypic and phenotypic heterogeneity in cell systems

Simon Syga, Josué Manik Nava-Sedeño and Andreas Deutsch

1 Mathematical definition of LGCA

A lattice-gas cellular automaton, like any true cellular automaton, is defined by a tuple $(\mathcal{L}, \mathcal{E}, \mathcal{R})$, where:

- $\mathcal{L} \subset \mathbb{R}^d$ is a d-dimensional spatial lattice with nodes $\mathbf{r} \in \mathcal{L}$.
- $\mathcal{E}$ is the state space, describing the set of possible values any lattice site may have.
- $\mathcal{R}$ is the transition rule that updates the states of all lattice sites, depending on a local set of lattice sites, or neighborhood $\mathcal{N} \subset \mathcal{L}$. The successive application of the rule simulates the passage of time.

1.1 Lattice

The model is defined on a regular d-dimensional spatial lattice, where each node $\mathbf{r}$ has b nearest neighbors, determined by the lattice geometry. For instance, in one dimension $b = 2$; in two dimensions, for a square lattice $b = 4$, and for a hexagonal lattice $b = 6$. Each node is connected to its nearest neighbors by a set of *velocity channels*, unit vectors pointing to the node's nearest neighbors. In two dimensions, they are represented by the unit vectors

$$\mathbf{c}_i = \begin{pmatrix} \cos \frac{2\pi i}{b} \\ \sin \frac{2\pi i}{b} \end{pmatrix}, \quad i = 0, 1, \dots, b-1. \tag{1}$$

Additionally, nodes may contain $a \in \mathbb{N}_0$ rest channels corresponding to zero velocity. These represent the possibility that a cell may not move at a certain time point.

1.2 State Space

Each node is assigned a state $\mathbf{s} \in \mathcal{E}$, $\mathcal{E} = \mathcal{O}^C$, $C = a + b$, where $\mathcal{O}$ is the channel occupation set, and represents the kinds of cells moving in the direction of a channel. In classic LGCA, the occupation set is Boolean, $\mathcal{O} = \{0, 1\}$, meaning that each channel can either be occupied by at most a single cell or be empty. In other words, cells follow an *exclusion principle*, similar to Fermions in quantum physics following the Pauli principle. The state of a node is thus given by a vector $\mathbf{s} = (s_0, s_1, \dots, s_{C-1})^T \in \{0, 1\}^C$. The total number of cells at a node is given by $n(\mathbf{s}) = \sum_{i=0}^{C-1} s_i$. The change in node states is determined by the states of nodes in its local neighborhood. We denote the neighborhood by $\mathcal{N}$. The most commonly used neighborhood is the von Neumann neighborhood $\mathcal{N}_N$ consisting of a node plus its direct neighbors, though other possibilities exist. We denote the state of the nodes in the neighborhood by $\mathbf{s}_{\mathcal{N}} \in \mathcal{E}^{|\mathcal{N}|}$, where $|\mathcal{N}|$ is the cardinality of the neighborhood. For example, $|\mathcal{N}_N| = 5$ on the two-dimensional square lattice, due to the four nearest neighbors and the central node.

1.3 Dynamics

The time evolution of LGCA is defined in discrete time steps $k \in \mathbb{N}$ by the parallel application of the rule $\mathcal{R}$ to every node in the lattice $\mathcal{L}$. In LGCA, the rule $\mathcal{R}$ consists of two steps: first, a typically stochastic interaction step $\mathcal{I}$ that incorporates the biological, or physical, mechanisms of interest, and second, a deterministic transport step $\mathcal{T}$ The *configuration* at a node $\mathbf{r}$ at the time step k represents the state of the lattice, i.e. the states and velocities of all cells, and is given

by the stochastic process $\boldsymbol{\eta} : \mathcal{L} \times \mathbb{N} \mapsto \mathcal{E}$. Similarly, the *neighborhood configuration* contains the information of the states of all nodes in a certain neighborhood, and is defined as

$$\boldsymbol{\eta}_{\mathcal{N}}(\mathbf{r}, k) := (\boldsymbol{\eta}(\mathbf{r}', k))_{\mathbf{r}' \in \mathcal{N}(\mathbf{r})} \in \mathcal{E}^{|\mathcal{N}|}. \tag{2}$$

During the interaction step, a post-interaction configuration $\boldsymbol{\eta}'(\mathbf{r}, k)$ is obtained given the Markovian operator $\mathcal{I}(\mathbf{s}_{\mathcal{N}}) : \mathcal{E}^{|\mathcal{N}|} \mapsto \mathcal{E}$ to update the node configuration $\boldsymbol{\eta}(\mathbf{r}, k) = \mathbf{s} \to \boldsymbol{\eta}'(\mathbf{r}, k) = \mathbf{s}'$ according to the transition probability $P(\mathbf{s} \to \mathbf{s}'|\mathbf{s}_{\mathcal{N}})$, which models all changes cells undergo due to interactions with other cells or the surrounding medium. Note that this is a conditional probability that depends on the states of the nodes in the neighborhood (which includes the state of the central node). Often, the interaction step is defined by the subsequent application of several stochastic transition rules. For example, imagine that we want to define a cell proliferation, death, and undirected movement model. Then we could define the interaction step as the subsequent application of a birth-and-death operator that adds and removes cells, and a migration operator that redistributes them within the node according to a rule that preserves cell number.
The transition probability of number-conserving interactions is typically defined as

$$P(\mathbf{s} \to \mathbf{s}' \mid \mathbf{s}_{\mathcal{N}}) = \frac{\delta(n(\mathbf{s}'), n(\mathbf{s}))}{Z(\mathbf{s}', \mathbf{s}_{\mathcal{N}})} \exp\left[E(\mathbf{s}', \mathbf{s}_{\mathcal{N}})\right], \tag{3}$$

where $E(\mathbf{s}', \mathbf{s}_{\mathcal{N}})$ is a pseudo-energy function assigning weights to post-interaction states modeling biological and/or physical mechanisms, and the Kronecker delta $\delta(n(\mathbf{s}'), n(\mathbf{s}))$ enforces mass conservation, by precluding configurations with more, or less, cells. We denote the state of the nodes in the neighborhood by $\mathbf{s}_{\mathcal{N}} \in \mathcal{E}^{|\mathcal{N}|}$.

In the transport step, particles move to adjacent nodes along their velocity channels. The dynamics of the LGCA are then summarized by the equation

$$\eta_i(\mathbf{r}, k+1) = \eta_i'(\mathbf{r} - \mathbf{c}_i, k), \tag{4}$$

where $\eta_i(\mathbf{r}, k)$ represents the occupation number of channel i at node $\mathbf{r}$ and time step k. The stochastic finite difference equation, is known as the *microdynamical equation*.

When considering finite space, boundary conditions must also be specified, such as periodic, reflective, or absorbing boundaries, which dictate the behavior of the LGCA at the lattice edges. These should agree with the experimental setup of the original biological system.

2 Evolutionary lattice-gas cellular automata

2.1 Concept

If tracking individual cells is needed, or if cells whould have distinct properties, IB-LGCA seems like a suitable framework at first. However, the exclusion principle of IB-LGCA may have undesired effects: Rules may need to be applied such that they restrict the movement of cells, and that the intermixing of cells can then be limited even on the long time scales of evolution. To do that, cells must be free to switch to the rest channel even at cell numbers approaching the node capacity C. The main idea of evolutionary LGCA (evo-LGCA) is to assign each channel *a finite set of elements* from the set of properties. Each element of the set in a channel indicates the individual property of a cell that resides in the channel.

2.2 State space

As in IB-LGCA, in evolutionary LGCA (evo-LGCA), each individual cell j is represented by its properties $p_j \in \mathcal{P}$, where $\mathcal{P}$ is a flexible set. For example, it could consist of individual labels $\mathcal{P} = \mathbb{N}$, continuous properties $\mathcal{P} = \mathbb{R}$, qualitative properties $\mathcal{P} = \{\text{cell type } 1, \text{cell type } 2, \text{cell type } 3\}$ or any combination of them. In general, two cells i, j can have the same property, i. e., $p_i = p_j$. Therefore, the population of cells is represented using a *multiset*, sometimes referred to as *bag*, $M : \mathcal{P} \mapsto \mathbb{N}_0$. $M(p)$ is the *multiplicity* of p, indicating how many cells in the population have the property p. The set of all possible cell populations given a property set $\mathcal{P}$, i. e., *all possible multisets on* $\mathcal{P}$, is denoted as $\mathbb{N}_0^{\mathcal{P}}$. The *support* of a multiset M is the set of relevant elements of M, i. e., elements with non-zero multiplicity, defined as $\mathrm{supp}(M) = \mathcal{S}(M) := \{p \in \mathcal{P} \mid M(p) \neq 0\}$. The set of finite cell populations with properties from $\mathcal{P}$ is defined as

$$\boxed{\mathcal{M}_{\mathcal{P}} := \left\{M \in \mathbb{N}_0^{\mathcal{P}} \mid |\mathcal{S}(M)| < \infty\right\}.} \tag{5}$$

In evo-LGCA, the state of every channel j is a finite cell population $s_j \in \mathcal{O} := \mathcal{M}_\mathcal{P}$. Consequently, the state space of evo-LGCA is $\mathcal{E} = \mathcal{O}^C = \mathcal{M}_\mathcal{P}^C$, where $C := b + a$ is the number of channels, b is the number of velocity channels, and a is the number of rest channels. The state of a node is a tuple $\mathbf{s} \in \mathcal{E}$. Usually, in evo-LGCA, as in BLGCA, the number of rest channels is either zero or one, i. e., $a \in \mathbb{B}$. The natural way to indicate cell absence in evo-LGCA is the empty multiset $M_0 := \{\} = \emptyset \in \mathcal{M}_\mathcal{P}$, which is always possible, regardless of the choice for the property set $\mathcal{P}$.

Consider a simple example, where $\mathcal{P} = \{\alpha_1, \alpha_2\} \subset [0, 1]$. α_1, α_2 could represent the probability that a cell will divide in one time step. Then the set of finite cell populations on $\mathcal{P}$ is

$$\mathcal{M}_\mathcal{P} = \{\emptyset, \{\alpha_1\}, \{\alpha_2\}, \{\alpha_1, \alpha_1\}, \{\alpha_2, \alpha_2\}, \{\alpha_1, \alpha_2\}, \{\alpha_1, \alpha_1, \alpha_2\}, \dots\}$$

for any finite multiplicity of $\alpha_1, \alpha_2 \in \mathcal{P}$.

Given these definitions, the number of cells in channel j in state s_j, denoted by $n(s_j)$, is equal to the cardinality of s_j, $n(s_j) := |s_i| < \infty$. This quantity is known as *channel occupation number*, or in short *channel occupation*, in analogy to LGCA. Consequently, the total number of cells on a node (*node occupation*) in state $\mathbf{s}$ is given by the sum of the cardinalities of the channel states $s_i \in \mathcal{M}_\mathcal{P}$

$$n(\mathbf{s}) := \sum_{i=0}^{C-1} |s_i| < \infty, \tag{6}$$

which is a finite number, because all s_i are finite multisets. The *cell population of a node* in state $\mathbf{s}$, $s_{\text{tot}}(\mathbf{s})$, is again a multiset, which is obtained by summation of the channel populations $s_i \in \mathcal{M}_\mathcal{P}$

$$\boxed{s_{\text{tot}}(\mathbf{s}) := \sum_{i=0}^{C-1} s_i.} \tag{7}$$

The sum of two cell populations $a + b$, where $a, b \in \mathcal{M}_\mathcal{P}$, is the multiset $c \in \mathcal{M}_\mathcal{P}$ defined by its multiplicity

$$M_c(p) = M_a(p) + M_b(p) \quad \forall p \in \mathcal{P}. \tag{8}$$

Similarly, the difference of two cell populations $a - b$, where $a, b \in \mathcal{M}_\mathcal{P}$, is defined as $c \in \mathcal{M}_\mathcal{P}$ with the multiplicity

$$M_c(p) = \max(M_a(p) - M_b(p), 0) \quad \forall p \in \mathcal{P}. \tag{9}$$

The definitions of sum and difference of cell populations extend to node states by point-wise application, i. e., $\mathbf{c} = \mathbf{a} \pm \mathbf{b}$, with $\mathbf{a}, \mathbf{b}, \mathbf{c} \in \mathcal{E}$, is defined as $\mathbf{c} := (a_0 \pm b_0, a_1 \pm b_1, \dots, a_{C-1} \pm b_{C-1})^T$.

Given the state space $\mathcal{E} := \mathcal{M}_\mathcal{P}^C$, the neighborhood state $\mathbf{s}_\mathcal{N}$, the node configuration $\boldsymbol{\eta} : \mathcal{L} \times \mathbb{N} \mapsto \mathcal{E}$ and the neighborhood configuration $\boldsymbol{\eta}_\mathcal{N} : \mathcal{L} \times \mathbb{N} \mapsto \mathcal{E}^{|\mathcal{N}|}$ are defined in complete analogy to classical LGCA.

2.3 Dynamics

2.3.1 Mass and property-conserving interactions

To define interactions that conserve the number of cells and their properties for evo-LGCA, similar methods to those for BLGCA can be used. In particular, if one assumes that each cell is assigned a new channel independently, individual channel transition probabilities can be used $T_i(p, \mathbf{s}_\mathcal{N})$, where $p \in \mathcal{P}$ are the properties of an individual cell. Then, the transition probability is a product of multinomial probability masses, i. e.,

$$\boxed{P(\mathbf{s} \to \mathbf{s}' \mid \mathbf{s}_\mathcal{N}) = \prod_{p \in \mathcal{S}(s_{\text{tot}}(\mathbf{s}))} M_{\text{tot}}(p)! \prod_{i=0}^{C-1} \frac{T_i^{M_i'(p)}(p, \mathbf{s}_\mathcal{N})}{M_i'(p)!} \delta(M'_{\text{tot}}(p), M_{\text{tot}}(p)).} \tag{10}$$

Here, the shorthand notation $M_i'(p) := M_{s_i'}(p)$ was used for the multiplicity of property p in channel i after the interaction step and $M_{\text{tot}}(p) := M_{s_{\text{tot}}(\mathbf{s})}(p), M'_{\text{tot}}(p) := M_{s_{\text{tot}}(\mathbf{s}')}(p)$ are the multiplicities of property p in the cell population of the node before and after interaction, respectively. Channel probabilities $T_i(p, \mathbf{s}_\mathcal{N}) : \mathcal{P} \times \mathcal{E}^{|\mathcal{N}|} \mapsto [0, 1]$ represent the probability that a cell with property p is assigned a channel i during the interaction step. When the interaction is mass and property-conserving, we have $\sum_{i=0}^{C-1} T_i(p, \mathbf{s}_\mathcal{N}) = 1$ for all $p \in \mathcal{P}$. We can interpret the transition probability in the following way: all cells with the same property are distributed to new channels using a multinomial distribution, like in BLGCA. Since this occurs independently for each property,

the multinomial probabilities are multiplied for each property p present in the node before the interaction, i.e., $\forall p \in \mathcal{S}(s_{\text{tot}}(\mathbf{s}))$. Such a rule is sometimes termed *rearrangement*, as it only rearranges the cells within the node but does not remove or add any cells or change their properties.

Note that this definition of transition probability coincides with mass-conserving interactions in the BLGCA framework if all cells are represented by the same property p. Then, the first product operator vanishes (since there is only one property to consider), the dependence of the channel probability T_i on the property p can be omitted by treating it as a fixed parameter, the multiplicities $M_{s'_i}$ can be identified with the occupation numbers of channel i.

For example, consider a transition probability that models an unbiased random walk. As in BLGCA the corresponding channel probability is $T_i = 1/C$ (if there is no additional weight for the rest channel). For applications for which tuning of the migratory activity of cells is important, a rest channel weight $\gamma \in \mathbb{R}$ can be used. The channel probabilities are then $T_i = 1/Z$ for $0 \leq i < b$, $T_i = Z^{-1} \exp \gamma$ for $i \geq b$ and Z is a normalization constant $Z := b + \exp \gamma$. In this case, the channel probabilities are constant and do not depend on the cell property p or the neighborhood state $\mathbf{s}_{\mathcal{N}}$. It is possible to extend the interaction by defining the rest channel weight as a function of the cell property $\gamma : \mathcal{P} \mapsto \mathbb{R}$ to study cells with varying tendencies to move.

2.3.2 Birth and death interactions

To study evolutionary dynamics, it is vital to define interaction steps that incorporate the births and deaths of cells. The interaction step can be then constructed as a combination of operators representing births and deaths, followed by mass and property-conserving interactions.

Death operator A death operator $D : \mathcal{E} \mapsto \mathcal{E}$ that models the independent death of each cell with a constant probability $\delta \in [0, 1]$, independent of the properties of the cell, can be defined as follows. First, consider the effect of the death operator on a single channel. Assume the death operator removes every property $p \in s_i \in \mathcal{M}_{\mathcal{P}}$ independently with probability δ. If every property would occur at most once in the channel state s_i, then the probability of a post-death state s'_i would be $P(s'_i \mid s_i) = \prod_{p \in s_i} (1-\delta)^{M'_i(p)} \delta^{M_i(p) - M'_i(p)}$. Here, the short-hand notation for the multiplicity of property p in state s_i from above $M'_i(p) = M_{s'_i}(p), M_i(p) = M_{s_i}(p)$ was used. We would keep every property with probability $(1 - \delta)$ and remove it with probability δ. However, as every property may be present more than once, the multiplicity of a property after applying the death operator follows a binomial distribution $B(k \mid p, n)$. This is because the survival/removal of each occurrence of the property p occurs in an individual Bernoulli trial. That means that every cell in every node can die independently of the others and the number of death events in every node is not limited. Lastly, since every occurrence of a property is handled independently, the outcomes of all channels are independent of each other. When all this is taken together, the transition probability of the death operator is

$$P(\mathbf{s}' \mid \mathbf{s}) = \prod_{i=0}^{C-1} \prod_{p \in \mathcal{S}(s_i)} \binom{M_i(p)}{M'_i(p)} (1 - \delta)^{M'_i(p)} \delta^{M_i(p) - M'_i(p)}. \tag{11}$$

The first product accounts for the independent outcomes for each channel, and the second product handles the independent outcomes for each relevant property.[1]

An equivalent way to define the death operator is to define a multiset of dying cells $\mathbf{s}_D$ with

$$P_D(\mathbf{s}_D \mid \mathbf{s}) = \prod_{i=0}^{C-1} \prod_{p \in \mathcal{S}(s_i)} \binom{M_{s_i}(p)}{M_{s_{i,D}}(p)} \delta^{M_{s_{i,D}}(p)} (1 - \delta)^{M_{s_i}(p) - M_{s_{i,D}}(p)} \tag{12}$$

Then the post-interaction state is equal to the difference of the pre-interaction state and the set of dying cells $\mathbf{s}' = \mathbf{s} - \mathbf{s}_D$. From the probability $P_D(\mathbf{s}_D \mid \mathbf{s})$, we also see that it is straightforward to define a death operator that depends on the cell properties. In that case, we can define the death probability as a function $\delta : \mathcal{P} \mapsto [0, 1]$ and plug it into the above transition probability.

Birth operator To define a birth operator, the same philosophy as for the death operator can be followed. First, one defines a new node population $\mathbf{s}_B$ of newly born cells, which are then added to the existing cells. To obtain $\mathbf{s}_B$, the following assumptions can be made:

[1] To apply the death operator defined above in a computer simulation, it is more efficient to loop over all cell properties in each channel state and remove them individually with probability δ, explicitly performing the individual Bernoulli trials. This is because the number of possible post-death states s'_i is usually very large and therefore it is not feasible to compute all transition probabilities .

1. In each time step, each cell with property $p \in \mathcal{P}$ proliferates with a probability $\alpha_i(p) \in [0, 1]$ that might depend on the channel i that it resides in.

2. The properties of the new cells $p' \in \mathcal{P}$ can be different from those of the already present cells. Therefore, one needs to define $p'(p), p' : \mathcal{P} \mapsto \mathcal{P}$, the property of the daughter cells in dependence on the property of the mother cell.

3. Newly born cells must be assigned to a channel according to the probabilities of the channels $T_i \in [0, 1], i = 0, 1, \ldots, C-1$, with $\sum_{i=0}^{C-1} T_i = 1$.

For simplicity, it is assumed that these three steps are independent. Note that there might be applications where it is necessary to revoke this assumption.

Typically, the property of a daughter cell p' is a random variable. Depending on the nature of the set of properties $\mathcal{P}$, an appropriate probability mass $f_{p',p} \in [0, 1]$, with $\sum_{p' \in \mathcal{P}} f_{p',p} = 1$, or probability density $f(p', p) \in \mathbb{R}_0^+$, with $\int_{\mathcal{P}} f(p', p)\,\mathrm{d}p' = 1$ can be chosen.

The multistep nature of the birth process makes it challenging to define a closed-form solution for the probability of the population of newly born cells $\mathbf{s}_B$. In practice, it is easier to loop over all cells in the node state $\mathbf{s}$, check if it proliferates with probability $\alpha_i(p)$, and if so, draw a property $p' \in \mathcal{P}$ for the daughter cell from a given probability distribution $f(p', p)$, and finally choose a channel for the daughter cell according to the channel probabilities $T_i, i = 0, \ldots, C-1$.

2.3.3 Coupling of interactions

Several biological mechanisms must be coupled in most applications, especially birth-and-death operators and mass-conserving interactions. To do that, defining the interaction step $\mathcal{I}$ as multiple substeps is practical. The most common example is applying a birth-and-death process, followed by a mass-conserving rule. Then the interaction step looks like

$$\mathbf{s} \underset{\text{Births \& deaths}}{\longrightarrow} \mathbf{s} + \mathbf{s}_B - \mathbf{s}_D \underset{\text{Rearrangement}}{\longrightarrow} \mathbf{s}'. \tag{13}$$

3 Mean-field approximation

To predict the local (nonspatial) dynamics of the cell density with proliferation rate α, $\psi(\alpha, t) = \langle n(\alpha, t) \rangle / K$, a mean-field approximation is used. The mean-field approximation neglects correlations between all random variables, such as cell numbers with different proliferation rates $n(\alpha, k), n(\alpha', k)$ and approximates the mean value of any function $f(X)$ of a random variable X by the function evaluated at the mean value of the random variable, i. e., $\langle f(X) \rangle \approx f(\langle X \rangle)$.

The number of cells with proliferation rate α at time step k changes during the interaction step as

$$n(\alpha, k+1) = n(\alpha, k) - \underbrace{\beta(k, n(\alpha, k))}_{\text{deaths}} + \underbrace{\pi(\boldsymbol{\eta}(k), k)}_{\text{births}}. \tag{14}$$

Here, $\beta(k, n(\alpha, k)) \in \{0, 1, \ldots, n(\alpha, k)\}$ is a stochastic variable that describes cell death. As all cells die with a constant probability δ in each time step, $\beta(n(\alpha), k)$ follows a Bernoulli distribution with the parameters $\delta, n(\alpha)$ and has the expected value

$$\langle \beta(k, n(\alpha, k)) \rangle = \sum_{n(\alpha,k)=0}^{\infty} \delta n(\alpha, k) P(n(\alpha, k)) = \delta \langle n(\alpha) \rangle. \tag{15}$$

The stochastic variable $\pi(\boldsymbol{\eta}(k), k)$ models cell proliferation. For simplicity, mutation terms are neglected ($p_\mu \approx 0$) to obtain

$$\langle \pi(n(\alpha, k), k) \rangle = \alpha \langle n(\alpha, k) \rangle \left(1 - \frac{\langle n_{\text{tot}}(k) \rangle}{K}\right), \tag{16}$$

where $n_{\text{tot}}(k) := s_{\text{tot}}(\boldsymbol{\eta}(k))$ is the total cell number at time step k.

Using a small time step length $\tau > 0$, one can introduce the continuous-time variable $t = k\tau$ and derive a differential equation for the cell density $\psi(\alpha, t) := \frac{\langle n(\alpha,t) \rangle}{K}$. With the results for the expected values of the stochastic variables describing cell death and proliferation, the expected value of the cell density is

$$\psi(\alpha, t+\tau) = \psi(\alpha, t) - \delta\psi(\alpha, t) + (1 - \Psi(t))\alpha\psi, \tag{17}$$

where $\Psi(t) := \langle n_{\mathrm{tot}}(t) \rangle / K = \int \psi(\alpha, t) \, \mathrm{d}\alpha$ is the normalized total cell density. Next, defining the death and proliferation rates δ', α', such that $\delta = \delta' \tau, \alpha = \alpha' \tau$, one can approximate the cell density by its Taylor approximation up to first order $\psi(\alpha, t + \tau) \approx \psi(\alpha, t) + \tau \partial_t \psi(\alpha, t)$. After rearranging terms, the following differential equation is obtained

$$\boxed{\frac{\mathrm{d}\psi}{\mathrm{d}t} = -\delta\psi + (1 - \Psi)\alpha\psi,} \tag{18}$$

where the prime symbols and the dependence of ψ on α and t have been dropped for better readability. Integrating the equation, an ordinary differential equation for the total cell density is obtained

$$\boxed{\frac{\mathrm{d}\Psi}{\mathrm{d}t} = -\delta\Psi + \langle \alpha \rangle (t)(1 - \Psi)\Psi,} \tag{19}$$

with the definition of the mean fitness value $\langle \alpha \rangle (t) := \frac{1}{\Psi(t)} \int \alpha \psi(\alpha, t) \, \mathrm{d}\alpha$. It is immediate that the total cell mass grows according to a logistic growth law with the mean proliferation rate $\langle \alpha \rangle (t)$ that changes over time due to evolutionary dynamics (which were neglected in this derivation). This is important because it shows that logistic growth laws (and other more complicated growth laws relevant in the context of cancer, like the Gompertz law) are still applicable even in heterogeneous populations.

Like the scaling of discrete to continuous time, continuous space $x = \epsilon r$ is introduced with the lattice spacing $\epsilon > 0$. It is important to note that all cells perform an unbiased random walk independently of other cells and their proliferation rate. Therefore, a diffusion equation in the macroscopic limit with diffusive scaling can describe the cell migration process, which can be derived with standard methods [1, 2]. Thus, the time evolution of the cell density $\psi(x, \alpha, t)$ in one spatial dimension x can be approximated by the PDE

$$\partial_t \psi = D \partial_x^2 \psi + \text{growth terms} + \text{mutation terms}, \tag{20}$$

where the exact form of the growth and mutation terms depends on the applied approximations, as discussed. Crucially, cells with different proliferation rates are only coupled locally at the same point x by growth and mutation terms. Consequently, the time evolution of the total cell mass in space can be obtained by integrating over all proliferation rates α

$$\partial_t \Psi = D \partial_x^2 \Psi + \langle \alpha \rangle \Psi (1 - \Psi) - \delta \Psi. \tag{21}$$

This is a generalized Fisher-KPP equation with variable proliferation rate $\langle \alpha \rangle (x, t)$ that depends on space and time via the fitness distribution $\psi(x, \alpha, t)$.

References

[1] A. Deutsch and S. Dormann, *Cellular Automaton Modeling of Biological Pattern Formation*. Boston: Birkhäuser, 2018.

[2] A. Deutsch, J. M. Nava-Sedeño, S. Syga, and H. Hatzikirou, "BIO-LGCA: A cellular automaton modelling class for analysing collective cell migration," *PLOS Comput. Biol.*, vol. 17, p. e1009066, June 2021.
